# Preterm birth and risk of type 1 and type 2 diabetes: a national cohort study

**DOI:** 10.1007/s00125-019-05044-z

**Published:** 2019-12-05

**Authors:** Casey Crump, Jan Sundquist, Kristina Sundquist

**Affiliations:** 1 Department of Family Medicine and Community Health, Icahn School of Medicine at Mount Sinai, One Gustave L. Levy Place, Box 1077, New York, NY 10029 USA; 2 Department of Population Health Science and Policy, Icahn School of Medicine at Mount Sinai, New York, NY USA; 3 Center for Primary Health Care Research, Lund University, Clinical Research Centre (CRC), building 28, floor 11, Jan Waldenströms gata 35, Skåne University Hospital, SE-205 02 Malmö, Sweden

**Keywords:** Adult, Diabetes mellitus, type 1, Diabetes mellitus, type 2, Gestational age, Infant, small for gestational age, Premature birth, Preterm birth

## Abstract

**Aims/hypothesis:**

Preterm birth (gestational age <37 weeks) has been associated with insulin resistance early in life. However, no large population-based studies have examined risks of type 1 and type 2 diabetes and potential sex-specific differences from childhood into adulthood. Clinicians will increasingly encounter adults who were born prematurely and will need to understand their long-term risks. We hypothesised that preterm birth is associated with increased risks of type 1 and type 2 diabetes into adulthood.

**Methods:**

A national cohort study was conducted of all 4,193,069 singletons born in Sweden during 1973–2014, who were followed up for type 1 and type 2 diabetes identified from nationwide diagnoses and pharmacy data to the end of 2015 (maximum age 43 years; median age at the end of follow-up 22.5 years). Cox regression was used to adjust for potential confounders, and co-sibling analyses assessed the influence of shared familial (genetic and/or environmental) factors.

**Results:**

In 92.3 million person-years of follow-up, 27,512 (0.7%) and 5525 (0.1%) people were identified with type 1 and type 2 diabetes, respectively. Gestational age at birth was inversely associated with both type 1 and type 2 diabetes risk. Adjusted HRs for type 1 and type 2 diabetes at age <18 years associated with preterm birth were 1.21 (95% CI, 1.14, 1.28) and 1.26 (95% CI, 1.01, 1.58), respectively, and at age 18–43 years were 1.24 (95% CI, 1.13, 1.37) and 1.49 (95% CI, 1.31, 1.68), respectively, compared with full-term birth. The associations between preterm birth and type 2 (but not type 1) diabetes were stronger among females (e.g. at age 18–43 years, females: adjusted HR, 1.75; 95% CI, 1.47, 2.09; males: 1.28; 95% CI, 1.08, 1.53; *p* < 0.01 for additive and multiplicative interaction). These associations were only partially explained by shared genetic or environmental factors in families.

**Conclusions/interpretation:**

In this large national cohort, preterm birth was associated with increased risk of type 1 and type 2 diabetes from childhood into early to mid-adulthood. Preterm-born children and adults may need early preventive evaluation and long-term monitoring for diabetes.

**Electronic supplementary material:**

The online version of this article (10.1007/s00125-019-05044-z) contains peer-reviewed but unedited supplementary material, which is available to authorised users.

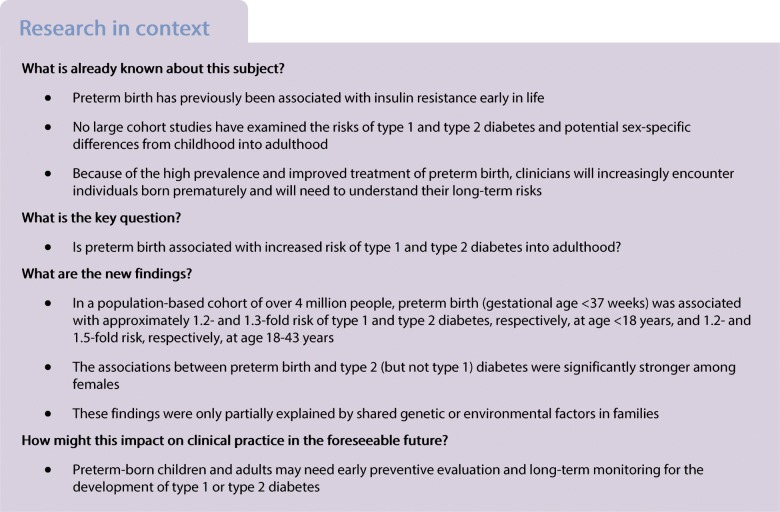



## Introduction

Diabetes is increasing in prevalence and now affects nearly 1 in 10 adults in the US [1] and worldwide [2]. In addition to family history and unhealthy lifestyle factors, early life exposures have been identified as potential risk factors for the development of diabetes later in life. According to Barker’s developmental origins theory, intrauterine nutritional abnormalities may permanently alter the body’s structure and metabolism, resulting in early life programming for future development of diabetes and other cardiometabolic disorders [3]. Recent evidence has suggested that developmental programming on the background of preterm birth may be particularly important for future outcomes [4–6]. Approximately 11% of all births worldwide [7] and 10% in the US [8, 9] occur preterm (gestational age < 37 weeks), and >95% of preterm infants in developed countries now survive into adulthood [6]. As a result, large numbers of preterm survivors (e.g. >400,000/year in the US) are now reaching adulthood (age ≥18) each year. Clinicians will increasingly encounter adult patients who were born preterm and will need to understand their long-term diabetes risks to guide preventive actions and anticipatory screening across the life course.

The largest prior studies of preterm birth and diabetes have focused on childhood (age <15 years) and reported increased risks of type 1 diabetes in preterm-born children [10–15]. A few smaller studies have also reported associations with type 2 diabetes in mid-adulthood [16–19]. However, to our knowledge, no large cohort studies have examined the risks of type 1 and type 2 diabetes and potential sex-specific differences from childhood into adulthood. Furthermore, it remains unclear whether such associations might be related to shared familial (genetic and/or environmental) factors that predispose to both preterm birth and diabetes, as opposed to direct effects of preterm birth.

To address these knowledge gaps, we conducted a national cohort study of over 4 million people in Sweden. The goals of this study were to examine associations between gestational age at birth and risk of type 1 or type 2 diabetes up to age 43 years, the maximum follow-up currently possible in this large cohort, to assess whether these associations differ according to sex or fetal growth, and to explore for potential confounding by shared familial (genetic and/or environmental) factors using co-sibling analyses. The results will help inform long-term monitoring, preventive actions and timely detection and treatment of diabetes in the growing population who were born prematurely.

## Methods

### Study population

The Swedish Birth Registry contains prenatal and birth information for nearly all births nationwide since 1973. Using this registry, we identified all 4,201,706 singleton live births in Sweden during 1973–2014. We excluded 8637 (0.2%) people who had missing information for gestational age, leaving 4,193,069 individuals (99.8% of the original cohort) for inclusion in the study. This study was approved by the ethics committee of Lund University in Sweden (No. 2010/476). Participant consent was not required as this study used only de-identified registry-based secondary data.

### Gestational age at birth ascertainment

Gestational age at birth was identified from the Swedish Birth Registry based on maternal report of last menstrual period in the 1970s and ultrasonography estimation starting in the 1980s and onward. This was analysed alternatively as a continuous variable or categorical variable with six groups: extremely preterm (22–28 weeks), very preterm (29–33 weeks), late preterm (34–36 weeks), early term (37–38 weeks), full-term (39–41 weeks, used as the reference group), and post-term (≥42 weeks). Early term birth (37–38 weeks) was examined as a separate category because it has previously been associated with increased risk of diabetes-related mortality relative to later term birth [6, 20]. In addition, the first three groups were combined to provide summary estimates for preterm birth.

### Diabetes ascertainment

The study cohort was followed up for the earliest diagnosis of type 1 or type 2 diabetes from birth through the end of follow-up in 2015 (maximum age 43 years; median age at end of follow-up 22.5 years). Type 1 diabetes was defined based on either of the following: (1) any ICD code specific for type 1 diabetes (ICD-9: 250.X1, 250.X3; ICD-10: E10) (for ICD-9 see www.icd9data.com/2007/Volume1; for ICD-10 see http://apps.who.int/classifications/icd10/browse/2016/en); or (2) any other ICD code for diabetes (ICD-8/9: 250; ICD-10: E11-E14) combined with insulin prescription (as described below) before age 30 years, consistent with prior epidemiological criteria [21]. Type 2 diabetes was defined based on ICD codes for diabetes (ICD-8/9: 250; ICD-10: E11-E14) that did not meet the additional criteria above for type 1.

All ICD codes were identified from primary or secondary diagnoses in the Swedish Hospital and Outpatient Registries. The Swedish Hospital Registry was started in 1964 and initially included all hospital discharge diagnoses from the Uppsala region of southern Sweden covering 16% of the national population, but was expanded to cover nearly 80% by 1973 (i.e. the beginning of the Swedish Birth Registry and the present study’s follow-up period) and >99% by 1987 [22, 23]. Diagnoses in this registry have been reported to have a positive predictive value of ~99% for diabetes [23, 24], although to our knowledge their ability to distinguish type 1 and type 2 has not been specifically evaluated. The Swedish Outpatient Registry contains all outpatient diagnoses from specialty clinics nationwide starting in 2001. The Swedish Pharmacy Registry includes all medication prescriptions nationwide since 1 July 2005, classified according to the Anatomic Therapeutic Chemical (ATC) System. Insulin prescriptions were identified based on any medication prescription with ATC code A10A.

### Other study variables

Other perinatal and maternal characteristics that may be associated with gestational age at birth and diabetes were identified using the Swedish Birth Registry and national census data, which were linked using an anonymous personal identification number [25–27]. The following were included as adjustment variables: birth year (continuous and categorical by decade), sex, birth order (1, 2, ≥3), maternal age at delivery (continuous), maternal education level (≤9, 10–12, >12 years), maternal birth country or region (Sweden, other Europe/US/Canada, Asia/Oceania, Africa, Latin America, other/unknown), maternal BMI (continuous), maternal smoking (0, 1–9, ≥10 cigarettes/day), diabetes during pregnancy (i.e. pregestational type 1 or type 2 or gestational diabetes as identified in the birth, hospital or outpatient registries; ICD-8: 250; ICD-9: 250, 648.0, 648.8; ICD-10: E10-E14, O24), preeclampsia (ICD-8: 637; ICD-9: 624.4–624.7; ICD-10: O14-O15), and other hypertensive disorders during pregnancy (ICD-8: 400–404; ICD-9: 401–405, 642.0–642.3, 642.9; ICD-10: I10-I15, O10-O11, O13, O15-O16).

Maternal BMI and smoking were assessed at the beginning of prenatal care starting in 1982, and were available for 61.1% and 74.2% of participants, respectively. Data were >99% complete for all other variables. Missing data for each covariate were imputed using a standard multiple imputation procedure based on the variable’s relationship with all other covariates and the outcome [28].

### Statistical analysis

Cox proportional hazards regression was used to compute HRs and 95% CIs for associations between gestational age at birth and incident type 1 or type 2 diabetes. These associations were examined from birth to age 43 years and in narrower age intervals (<18 or 18–43 years) among individuals living in Sweden without a prior diagnosis of the outcome at the beginning of the respective interval. The data were analysed as time-to-event with attained age as the Cox model time axis. To account for competing events that would preclude a diabetes diagnosis, individuals were censored at death as identified in the Swedish Death Registry (*n* = 41,485; 1.0%) or emigration as determined by absence of a Swedish residential address in census data (*n* = 258,834; 6.2%). Analyses were conducted both unadjusted and adjusted for covariates (as above). The proportional hazards assumption was assessed by examining log–log plots [29], and was met in each model.

Co-sibling analyses were performed to assess for potential confounding effects of unmeasured shared familial (genetic and/or environmental) factors among all individuals with at least one full sibling (*N* = 3,481,247; 83.0% of the cohort). This approach can help further elucidate whether associations observed in the primary analyses are due to direct effects of preterm birth as opposed to shared genetic or environmental factors that predispose to both preterm birth and diabetes. Relevant environmental factors within families may potentially include lifestyle exposures such as poor diet or physical inactivity. These analyses used stratified Cox regression with a separate stratum for each family as identified by the mother’s and father’s anonymous identification numbers. In the stratified Cox model, each set of siblings had its own baseline hazard function that reflects the family’s shared genetic and environmental factors, and thus associations between gestational age at birth and type 1 or type 2 diabetes were examined within families, controlling for their shared factors. In addition, these analyses were further adjusted for the same covariates as in the main analyses. For type 2 diabetes, co-sibling analyses were also performed after stratifying by sex.

Potential interactions between preterm birth and sex or fetal growth were examined in relation to type 1 or type 2 diabetes risk on the additive and multiplicative scale. Additive interactions were assessed using the ‘relative excess risk due to interaction’ (RERI), which is computed for binary variables as: RERI_HR_ = HR_11_ − HR_10_ − HR_01_ + 1. Multiplicative interactions were assessed using the ratio of HRs: HR_11_/(HR_10_ × HR_01_). A positive additive interaction is indicated if the RERI is >0, and a positive multiplicative interaction if the ratio of HRs is >1 [30, 31].

The following secondary analyses were also performed: (1) Associations were examined between fetal growth (small for gestational age [SGA; <10th percentile], appropriate for gestational age [AGA; 10th–90th percentile], large for gestational age [LGA; >90th percentile]) and type 1 or type 2 diabetes. (2) Type 1 and type 2 diabetes risks were explored after stratifying by spontaneous (71.4%) vs medically indicated (28.6%) preterm birth, which was systematically recorded starting in 1990 (*N* = 2,535,775 [60.5% of the cohort]; maximum age 26 years at end of follow-up). (3) As an alternative to multiple imputation, a sensitivity analysis was performed after restricting to participants without missing data (*N* = 2,561,146 [61.1% of the cohort]). All statistical tests were 2-sided and used an α-level of 0.05. All analyses were conducted using Stata version 15.1 (StataCorp, College Station, TX, USA).

## Results

Table 1 shows perinatal and maternal characteristics by gestational age at birth. Preterm infants were more likely than full-term infants to be male or firstborn; and their mothers were more likely to be at the extremes of age, have low education level, smoke, or have diabetes, preeclampsia or other hypertensive disorders during their pregnancy.

**Table 1:** Characteristics of study participants by gestational age at birth, Sweden, 1973–2014

	Extremely preterm	Very preterm	Late preterm	Early term	Full-term	Post-term
	(22–28 weeks)	(29–33 weeks)	(34–36 weeks)	(37–38 weeks)	(39–41 weeks)	(≥42 weeks)
	*n* = 11,591 (0.3%)	*n* = 41,106 (1.0%)	*n* = 157,342 (3.8%)	*n* = 740,391 (17.7%)	*n* = 2,896,444 (69.1%)	*n* = 346,195 (8.3%)
Child characteristics						
Sex						
Male	6288 (54.2)	22,975 (55.9)	85,570 (54.4)	381,140 (51.5)	1,471,368 (50.8)	188,358 (54.4)
Female	5303 (45.8)	18,131 (44.1)	71,772 (45.6)	359,251 (48.5)	1,425,076 (49.2)	157,837 (45.6)
Fetal growth						
SGA	294 (2.5)	5409 (13.2)	15,623 (9.9)	54,405 (7.3)	276,687 (9.6)	66,724 (19.3)
AGA	10,991 (94.8)	34,043 (82.8)	128,530 (81.7)	601,039 (81.2)	2,326,431 (80.3)	253,586 (73.2)
LGA	306 (2.6)	1654 (4.0)	13,189 (8.4)	84,947 (11.5)	293,326 (10.1)	25,885 (7.5)
Birth order						
1	5764 (49.7)	21,156 (51.5)	78,019 (49.6)	297,655 (40.2)	1,219,056 (42.1)	172,702 (49.9)
2	3311 (28.6)	11,598 (28.2)	47,050 (29.9)	271,130 (36.6)	1,087,624 (37.6)	111,055 (32.1)
≥3	2516 (21.7)	8352 (20.3)	32,273 (20.5)	171,606 (23.2)	589,764 (20.4)	62,438 (18.0)
Maternal characteristics						
Age (years)						
<20	512 (4.4)	1902 (4.6)	6476 (4.1)	22,069 (3.0)	84,019 (2.9)	12,962 (3.7)
20–24	2196 (18.9)	8336 (20.3)	33,190 (21.1)	139,115 (18.8)	580,841 (20.1)	76,287 (22.0)
25–29	3427 (29.6)	12,734 (31.0)	51,191 (32.5)	243,257 (32.9)	1,018,871 (35.2)	121,285 (35.0)
30–34	3148 (27.2)	10,954 (26.6)	41,555 (26.4)	211,859 (28.6)	821,668 (28.4)	92,813 (26.8)
35–39	1795 (15.5)	5718 (13.9)	20,167 (12.8)	100,963 (13.6)	330,847 (11.4)	36,883 (10.7)
≥40	513 (4.4)	1462 (3.6)	4763 (3.0)	23,128 (3.1)	60,198 (2.1)	5965 (1.7)
Education (years)						
≤9	1955 (16.9)	6768 (16.5)	24,405 (15.5)	104,171 (14.1)	367,829 (12.7)	48,594 (14.0)
10–12	5495 (47.4)	19,537 (47.5)	74,269 (47.2)	338,702 (45.7)	1,304,778 (45.0)	157,018 (45.4)
>12	4141 (35.7)	14,801 (36.0)	58,668 (37.3)	297,518 (40.2)	1,223,837 (42.3)	140,583 (40.6)
Birth country or region						
Sweden	8984 (77.5)	33,666 (81.9)	130,227 (82.8)	603,828 (81.6)	2,424,626 (83.7)	294,280 (85.0)
Other Europe/US/Canada	1171 (10.1)	3581 (8.7)	13,239 (8.4)	62,313 (8.4)	236,119 (8.2)	27,999 (8.1)
Asia/Oceania	826 (7.1)	2340 (5.7)	9108 (5.8)	50,443 (6.8)	150,854 (5.2)	11,962 (3.5)
Africa	339 (2.9)	723 (1.8)	2055 (1.3)	10,868 (1.5)	46,094 (1.6)	8025 (2.3)
Latin America	121 (1.0)	360 (0.9)	1522 (1.0)	8476 (1.1)	24,158 (0.8)	2014 (0.6)
Other/unknown	150 (1.3)	436 (1.1)	1191 (0.8)	4463 (0.6)	14,593 (0.5)	1915 (0.6)
BMI (kg/m^2^)						
<18.5	217 (1.9)	1069 (2.6)	4808 (3.1)	21,784 (2.9)	65,600 (2.3)	4649 (1.3)
18.5–24.9	8614 (74.3)	31,712 (77.2)	121,37 (77.1)	567,018 (76.6)	2,279,549 (78.7)	275,218 (79.5)
25.0–29.9	1910 (16.5)	5735 (14.0)	21,663 (13.8)	107,959 (14.6)	404,340 (14.0)	46,600 (13.5)
≥30.0	850 (7.3)	2590 (6.3)	9534 (6.1)	43,630 (5.9)	146,991 (5.1)	19,728 (5.7)
Smoking (cigarettes/day)						
0	8346 (72.0)	29,152 (70.9)	114,671 (72.9)	565,076 (76.3)	2,216,387 (76.5)	247,302 (71.4)
1–9	2557 (22.1)	9391 (22.8)	33,688 (21.4)	138,352 (18.7)	567,341 (19.6)	87,934 (25.4)
≥10	688 (5.9)	2563 (6.2)	8983 (5.7)	36,963 (5.0)	112,716 (3.9)	10,959 (3.2)
Diabetes during pregnancy	336 (2.9)	1552 (3.8)	6151 (3.9)	19,121 (2.6)	31,304 (1.1)	2260 (0.7)
Preeclampsia	1650 (14.2)	7251 (17.6)	16,095 (10.2)	39,382 (5.3)	94,720 (3.3)	11,833 (3.4)
Other hypertensive disorders	187 (1.6)	714 (1.7)	2241 (1.4)	8897 (1.2)	24,746 (0.9)	2347 (0.7)

Data are *n* (%)

### Associations between gestational age at birth and diabetes

In 92.3 million person-years of follow-up, 27,512 (0.7%) and 5525 (0.1%) individuals were identified with type 1 and type 2 diabetes, respectively. The median age at the end of follow-up was 22.5 years, and median ages at diagnosis were 14.9 years for type 1 and 29.2 years for type 2 diabetes. The type 1 incidence rate (per 100,000 person-years) was 29.80 in the overall cohort, 36.78 among those born preterm, and 28.80 among those born full-term. The corresponding incidence rates for type 2 diabetes were 5.98, 8.48 and 5.56, respectively.

Gestational age at birth was inversely associated with both type 1 and type 2 diabetes risk at age <18 years (adjusted HR per additional week of gestation, type 1: 0.96; 95% CI 0.95, 0.97; type 2: 0.95; 0.93, 0.98; Table 2). Adjusted HRs for type 1 and type 2 diabetes associated with preterm birth were 1.21 (95% CI, 1.14, 1.28) and 1.26 (1.01, 1.58), respectively. In contrast, children born extremely preterm had a lower risk of type 1 diabetes at age <18 years (adjusted HR, 0.51; 95% CI 0.32, 0.81) but a near-significantly higher risk of type 2 diabetes (2.21; 95% CI, 0.99, 4.95), compared with those born full-term (Table 2).

**Table 2:** Associations between gestational age at birth and risk of type 1 and type 2 diabetes, Sweden, 1973–2015

	All				Females			Males		
	Cases	Rate^a^	Risk difference (95% CI)^b^	Adjusted HR (95% CI)^c^	Cases	Rate^a^	Adjusted HR (95% CI)^c^	Cases	Rate^a^	Adjusted HR (95% CI)^c^
Attained ages <18 years										
Type 1 diabetes										
Preterm	1177	40.59	8.23 (5.85, 10.61)	1.21 (1.14, 1.28)	531	40.39	1.26 (1.15, 1.38)	646	40.75	1.17 (1.07, 1.26)
Extremely preterm	18	17.33	−15.03 (−23.05, −7.00)	0.51 (0.32, 0.81)	7	14.16	0.44 (0.21, 0.92)	11	20.22	0.57 (0.32, 1.03)
Very preterm	172	31.52	−0.83 (−5.58, 3.91)	0.93 (0.80, 1.08)	82	33.89	1.06 (0.85, 1.32)	90	29.64	0.84 (0.68, 1.04)
Late preterm	987	43.86	11.50 (8.71, 14.29)	1.30 (1.22, 1.39)	442	43.20	1.35 (1.22, 1.48)	545	44.41	1.27 (1.17, 1.39)
Early term	4060	38.31	5.95 (4.65, 7.25)	1.16 (1.12, 1.20)	1813	35.40	1.12 (1.06, 1.18)	2247	41.04	1.19 (1.14, 1.25)
Full-term	13,610	32.36	Reference	Reference	6360	30.72	Reference	7250	33.95	Reference
Post-term	1406	27.05	−5.31 (−6.82, −3.79)	0.86 (0.81, 0.91)	617	25.68	0.87 (0.80, 0.95)	789	28.24	0.85 (0.79, 0.91)
Per additional week (trend)				0.96 (0.95, 0.97)			0.96 (0.95, 0.97)			0.96 (0.95, 0.97)
Type 2 diabetes										
Preterm	89	3.07	0.93 (0.27, 1.58)	1.26 (1.01, 1.58)	48	3.65	1.60 (1.18, 2.17)	41	2.59	1.01 (0.73, 1.39)
Extremely preterm	6	5.78	3.63 (−0.99, 8.26)	2.21 (0.99, 4.95)	4	8.09	3.38 (1.26, 9.06)	2	3.68	1.32 (0.33, 5.28)
Very preterm	25	4.58	2.44 (−0.64, 4.24)	1.85 (1.24, 2.77)	13	5.37	2.33 (1.33, 4.07)	12	3.95	1.50 (0.84, 2.67)
Late preterm	58	2.58	0.44 (−0.24, 1.11)	1.07 (0.82, 1.40)	31	3.03	1.34 (0.92, 1.93)	27	2.20	0.87 (0.59, 1.28)
Early term	328	3.10	0.95 (0.59, 1.32)	1.33 (1.17, 1.51)	177	3.46	1.56 (1.31, 1.86)	151	2.76	1.13 (0.94, 1.36)
Full-term	901	2.14	Reference	Reference	421	2.03	Reference	480	2.25	Reference
Post-term	127	2.44	0.30 (−0.15, 0.75)	1.20 (0.99, 1.45)	46	1.91	1.02 (0.75, 1.38)	81	2.90	1.33 (1.05, 1.68)
Per additional week (trend)				0.95 (0.93, 0.98)			0.91 (0.88, 0.94)			0.99 (0.96, 1.03)
Attained ages 18–43 years										
Type 1 diabetes										
Preterm	427	29.22	7.30 (4.46, 10.14)	1.24 (1.13, 1.37)	168	25.77	1.34 (1.14, 1.57)	259	32.00	1.19 (1.05, 1.35)
Extremely preterm	19	49.66	27.74 (5.40, 50.08)	2.22 (1.41, 3.48)	12	64.48	3.55 (2.01, 6.26)	7	35.62	1.35 (0.64, 2.84)
Very preterm	78	29.25	7.33 (0.81, 13.85)	1.25 (1.00, 1.56)	28	23.89	1.24 (0.85, 1.80)	50	33.46	1.25 (0.95, 1.66)
Late preterm	330	28.54	6.62 (3.47, 9.76)	1.21 (1.08, 1.36)	128	24.80	1.28 (1.07, 1.53)	202	31.55	1.17 (1.01, 1.35)
Early term	1349	26.46	4.54 (3.00, 6.08)	1.17 (1.10, 1.24)	511	21.39	1.14 (1.04, 1.26)	838	30.93	1.18 (1.09, 1.28)
Full-term	4773	21.92	Reference	Reference	1934	18.11	Reference	2839	25.59	Reference
Post-term	710	21.93	0.01 (−1.72, 1.74)	1.00 (0.93, 1.08)	277	17.66	0.98 (0.86, 1.11)	433	25.96	1.02 (0.92, 1.12)
Per additional week (trend)				0.96 (0.95, 0.98)			0.96 (0.94, 0.98)			0.97 (0.95, 0.98)
Type 2 diabetes										
Preterm	281	19.23	7.08 (4.78, 9.37)	1.49 (1.31, 1.68)	142	21.78	1.75 (1.47, 2.09)	139	17.17	1.28 (1.08, 1.53)
Extremely preterm	11	28.75	16.60 (−0.40, 33.60)	2.55 (1.41, 4.62)	7	37.61	3.50 (1.67, 7.37)	4	20.36	1.74 (0.65, 4.63)
Very preterm	49	18.38	6.22 (1.06, 11.39)	1.42 (1.07, 1.88)	30	25.60	2.05 (1.43, 2.95)	19	12.71	0.95 (0.60, 1.49)
Late preterm	221	19.11	6.96 (4.40, 9.52)	1.47 (1.28, 1.69)	105	20.34	1.63 (1.33, 1.99)	116	18.12	1.35 (1.12, 1.63)
Early term	656	12.87	0.72 (−0.37, 1.80)	1.09 (1.00, 1.18)	308	12.89	1.14 (1.01, 1.30)	348	12.84	1.04 (0.92, 1.17)
Full-term	2646	12.15	Reference	Reference	1232	11.54	Reference	1414	12.74	Reference
Post-term	497	15.35	3.20 (1.77, 4.63)	1.10 (1.00, 1.21)	211	13.45	1.02 (0.88, 1.18)	286	17.14	1.17 (1.03, 1.33)
Per additional week (trend)				0.97 (0.95, 0.98)			0.94 (0.92, 0.96)			0.99 (0.97, 1.01)

^a^Incidence rate per 100,000 person-years

^b^Incidence rate difference per 100,000 person-years

^c^Adjusted for child characteristics (birth year, sex, birth order) and maternal characteristics (age, education, birth country or region, BMI, smoking, diabetes, preeclampsia, other hypertensive disorders during pregnancy)

Preterm, <37 weeks; extremely preterm, 22–28 weeks; very preterm, 29–33 weeks; late preterm, 34–36 weeks; early term, 37–38 weeks; full-term, 39–41 weeks; post-term, ≥42 weeks)

**Fig. 1:**
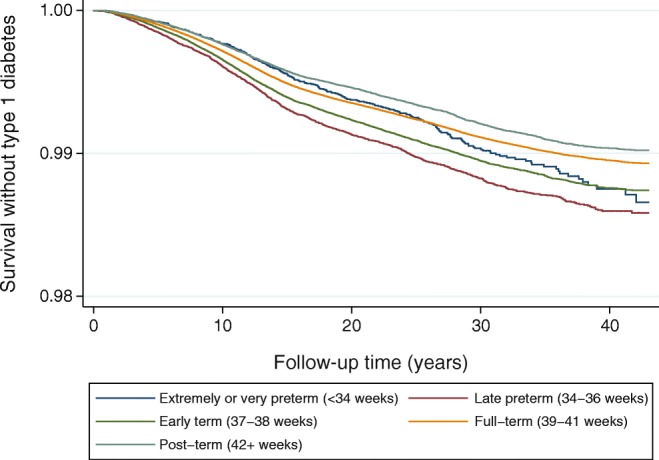
Kaplan–Meier curves for time to type 1 diabetes

**Fig. 2:**
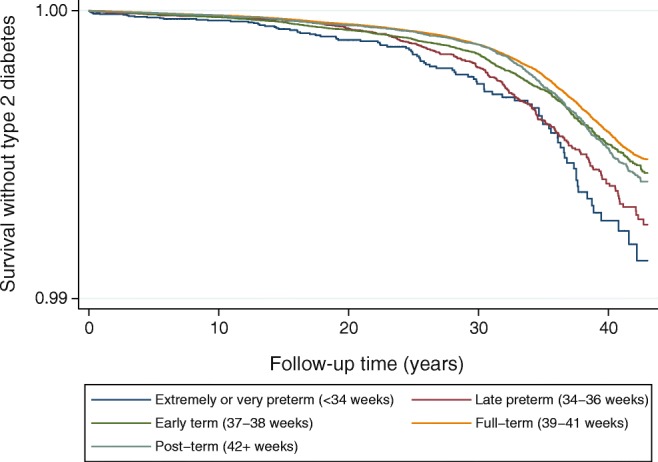
Kaplan–Meier curves for time to type 2 diabetes

Gestational age at birth also was inversely associated with new-onset type 1 and type 2 diabetes at age 18–43 years (adjusted HR per additional week of gestation, type 1: 0.96; 95% CI 0.95, 0.98; type 2: 0.97; 0.95, 0.98). The corresponding HRs comparing preterm vs full-term birth were 1.24 (95% CI 1.13, 1.37) and 1.49 (1.31, 1.68), respectively. Extremely preterm birth was associated with >2-fold risks of both type 1 (adjusted HR, 2.22; 95% CI 1.41, 3.48) and type 2 (2.55; 1.41, 4.62) diabetes. Preterm birth was more strongly associated with type 2 than type 1 diabetes in adulthood (*p*_heterogeneity_ < 0.001). Across all models, most adjusted HRs were <10% lower than unadjusted HRs (electronic supplementary material [ESM] Table 1). Kaplan–Meier curves for type 1 and type 2 diabetes by gestational age group are shown in Figs. 1 and 2.

### Sex-specific analyses

Significant interactions were found between preterm birth and sex in relation to type 2 but not type 1 diabetes (ESM Tables 2 and 3). Among individuals born full-term, type 2 diabetes incidence across all attained ages was slightly higher among males (5.84 per 100,000 person-years) than females (5.27). However, the opposite pattern was observed in those born preterm, with higher incidence among females (9.66) than males (7.52) (ESM Table 3). Preterm birth and female sex had a positive interaction on both the additive (*p* = 0.002) and multiplicative (*p* = 0.006) scale (i.e. their combined effect on type 2 diabetes risk was greater than the sum or product of their separate effects; ESM Table 3). The positive additive interaction indicates that preterm birth accounted for significantly more type 2 diabetes cases among females than males. In contrast, no interactions were found between preterm birth and sex in relation to type 1 diabetes on either the additive (*p* = 0.22) or multiplicative (*p* = 0.09) scale (ESM Table 2).

**Fig. 3:**
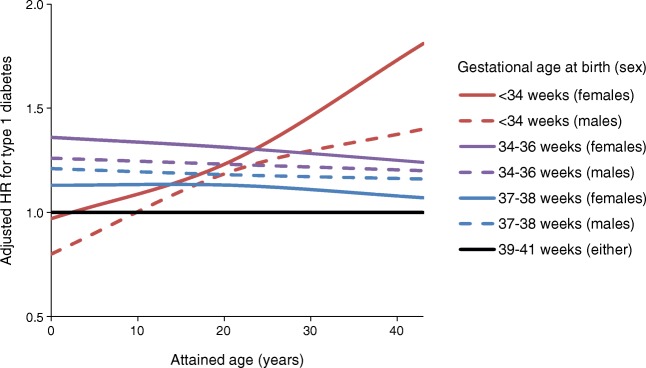
Adjusted HRs for new-onset type 1 diabetes by gestational age at birth compared with full-term birth, Sweden, 1973–2015

**Fig. 4:**
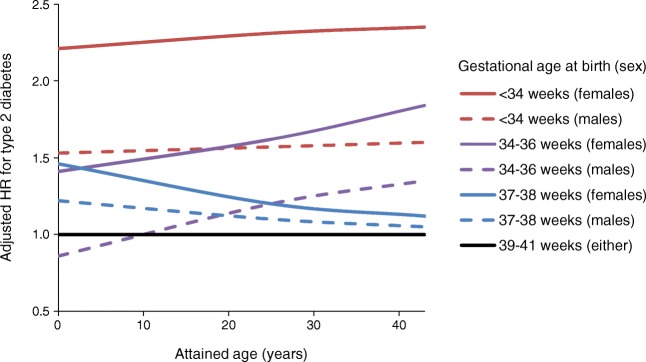
Adjusted HRs for new-onset type 2 diabetes by gestational age at birth compared with full-term birth, Sweden, 1973–2015

In sex-stratified analyses, preterm birth was associated with significantly increased risks of both type 1 and type 2 diabetes among females and males at all ages, except type 2 diabetes among males at ages <18 years (Table 2). Females born preterm had ~1.3-fold risks and males had ~1.2-fold risks of type 1 diabetes both at ages <18 and 18–43 years. For type 2 diabetes, adjusted HRs at age <18 years were 1.60 (95% CI 1.18, 2.17) among females and 1.01 (0.73, 1.39) among males, and at age 18–43 years were 1.75 (1.47, 2.09) among females and 1.28 (1.08, 1.53) among males. Females born extremely preterm had >3-fold risks of type 2 diabetes both at ages <18 and 18–43 years. Figs. 3 and 4 show adjusted HRs for type 1 and type 2 diabetes risk, respectively, by attained age for different gestational age groups.

### Co-sibling analyses

Co-sibling analyses to control for unmeasured shared familial factors resulted in partial attenuation of most risk estimates (ESM Table 4). At age <18 years, the adjusted HR for type 1 diabetes associated with preterm birth was 1.21 (95% CI 1.14, 1.28) in the primary analysis and 1.16 (1.04, 1.30) in the co-sibling analysis; the corresponding HRs for type 2 diabetes were 1.26 (1.01, 1.58) and 1.13 (0.75, 1.70; based on only 21 cases), respectively. At age 18–43 years, the adjusted HR for type 1 diabetes associated with preterm birth was 1.24 (95% CI 1.13, 1.37) in the primary analysis and 1.14 (0.92, 1.41) in the co-sibling analysis. In contrast, the corresponding HR for type 2 diabetes at age 18–43 years in the primary analysis (1.49; 95% CI 1.31, 1.68) was not attenuated in the co-sibling analysis (1.55; 1.12, 2.13).

After stratifying by sex, co-sibling analyses of type 2 diabetes yielded similar results. For example, the adjusted HR at age 18–43 years comparing preterm with full-term birth was 1.50 (95% CI 0.78, 2.86) among full sisters and 1.41 (0.75, 2.64) among full brothers.

### Secondary analyses

In analyses of fetal growth, SGA was a strong risk factor for type 2 but not type 1 diabetes, compared with AGA (e.g. age <18 years, type 1: adjusted HR, 0.88; 95% CI 0.83, 0.93; type 2: 1.61; 1.38, 1.89; age 18–43 years, type 1: 1.17; 1.09, 1.26; type 2: 1.79; 1.65, 1.93; see ESM Table 5 for complete results). The risk of type 2 diabetes was highest among individuals born both preterm and SGA (adjusted HR, 2.24; 95% CI 1.70, 2.96; relative to those born full-term and AGA). However, there was no evidence of interaction between preterm birth and SGA in relation to type 1 or type 2 diabetes risk on either the additive or multiplicative scale (type 1: *p* = 0.13 and *p* = 0.13; type 2: *p* = 0.81 and *p* = 0.52, respectively; ESM Tables 6 and 7). In a sensitivity analysis that was restricted to AGA births, all results were negligibly changed (e.g. preterm vs full-term, ages <18 years, type 1: adjusted HR, 1.20; 95% CI 1.12, 1.28; type 2: 1.24; 0.96, 1.60; ages 18–43 years, type 1: 1.24; 1.11, 1.39; type 2: 1.49; 1.29, 1.72).

Compared with full-term birth, both spontaneous and medically indicated preterm birth were associated with increased risks of type 1 diabetes (adjusted HR, 1.22; 95% CI 1.11, 1.34; and 1.22; 1.09, 1.37, respectively; *p* = 0.97 for difference in HRs) and type 2 diabetes (1.41; 1.05, 1.90; and 1.45; 1.02, 2.04, respectively; *p* = 0.92 for difference in HRs).

Restricting to participants without missing data resulted in modest changes in most risk estimates (e.g. preterm vs full-term, age <18 years, type 1 diabetes: adjusted HR, 1.22; 95% CI 1.13, 1.32; type 2 diabetes: 1.42; 1.08, 1.87; age 18–43 years, type 1: 1.07; 0.87, 1.31; type 2: 1.96; 1.48, 2.61).

## Discussion

In this large national cohort study, preterm birth was associated with increased risks of type 1 and type 2 diabetes from childhood into early to mid-adulthood. Preterm birth was associated with approximately 1.2- and 1.3-fold risk of type 1 and type 2 diabetes, respectively, at age <18 years, and 1.2- and 1.5-fold risk, respectively, at age 18–43 years. The associations between preterm birth and type 2 (but not type 1) diabetes were significantly stronger among females. Early term birth (37–38 weeks) was also associated with modestly increased risk of type 1 and type 2 diabetes from childhood into adulthood. Co-sibling analyses suggested that these findings were only partially explained by shared genetic or environmental factors in families.

Several prior studies have linked preterm birth with type 1 diabetes in childhood. A Swedish cohort study of 3.6 million children aged <15 years, who overlapped with the present cohort, reported 10–20% increased risk of type 1 diabetes among those born at 33–36 or 37–38 weeks [11]. However, consistent with our findings, infants born at <33 weeks had lower risk of type 1 diabetes during childhood compared with those born at term. To our knowledge, this finding remains unexplained and has not been assessed in other studies, and thus will need confirmation in other large independent cohorts. A UK cohort study of 3.8 million children aged <12 years reported 15–30% increased risk of type 1 diabetes among those born preterm or early term compared with full-term, but did not specifically examine earlier gestational ages [10]. An Australian cohort study of 558,633 children aged <15 years reported 1.4- and 1.2-fold risk among those born preterm or early term, respectively [13]. A matched cohort study in Taiwan with 37,119 preterm and 162,020 term children (mean age 8–9 years) reported 1.8- and 2.5-fold risks of type 1 and type 2 diabetes, respectively [14]. A Swedish cohort study of 630,090 adults aged 25–37 years found that those born preterm had modestly (10–25%) increased odds of medication prescription for diabetes, which was predominantly type 1 [32].

Several smaller studies have also reported associations between preterm birth and type 2 diabetes in mid-adulthood. For example, a Finnish cohort study of 12,813 adults aged >40 years reported a 1.6-fold (95% CI 1.00, 2.52) risk for those born at gestational age <35 vs 37–41 weeks [16]. A Swedish cohort study of 6425 adults aged 37–62 years reported that those born at <33 weeks had a 1.6-fold (95% CI 1.33, 2.11) risk of type 2 diabetes based on inpatient diagnoses [17]. A Scottish cohort study of 5973 adults aged 46–50 years found that preterm birth was associated with a 2-fold (95% CI 1.18, 3.53) risk of self-reported type 2 diabetes [18]. In the largest meta-analysis to date with ~2.2 million participants from 23 studies, the pooled ORs for association between preterm birth and type 1 or type 2 diabetes were 1.18 (95% CI 1.11, 1.25; based on 18 studies) and 1.51 (1.32, 1.72; 5 studies), respectively [33].

To our knowledge, no prior studies have examined gestational age at birth in relation to both type 1 and type 2 diabetes and potential sex-specific differences from childhood into adulthood. The present study addressed these gaps using nationwide diagnoses in the largest cohort to date, while controlling for multiple potential confounders. In this cohort and in other general populations, type 2 diabetes has a higher overall prevalence among men [1, 2]. However, we found that preterm birth was more strongly associated with type 2 diabetes and accounted for significantly more cases among women. To our knowledge, this sex-specific difference has not been previously reported and thus warrants confirmation in other well-powered studies. We found that most of the observed associations were partially explained by shared genetic or environmental factors in families. However, the association between preterm birth and type 2 diabetes in adulthood specifically appeared independent of shared familial factors.

These findings may have multiple underlying mechanisms that involve pancreatic beta cell function and insulin resistance. Preterm birth interrupts the development of pancreatic beta cells, which are formed predominantly in the third trimester of pregnancy, and might permanently reduce their number or function [34]. The limited available evidence for beta cell mass and function after preterm birth is conflicting. For example, experimental evidence has shown that induced preterm birth in sheep resulted in a 65% reduction of beta cell mass and reduced insulin secretory capacity that persisted into adulthood [35]. However, limited evidence from human studies has suggested that prematurity may be associated with appropriate insulin secretion consistent with normal beta cell function in early or mid-adulthood [36, 37]. Preterm birth also alters immune function including T cell response [38], which may potentially mediate its association with type 1 diabetes, consistent with its autoimmune aetiology [39]. Other contributing factors may include exposure to antenatal corticosteroids and rapid catch-up growth in infancy, leading to visceral adiposity and insulin resistance [40–42]. Iatrogenic factors from intensive care, including suboptimal nutrition and adverse effects of medications or procedures, may further impair glucose metabolism [4]. Several studies have reported reduced insulin sensitivity in preterm-born children or adults compared with term-born controls [42–44]. These associations are further modified by lifestyle factors across the life course, including diet, exercise and obesity [45], which are important targets for intervention.

Because of major advances in neonatal and paediatric care, most preterm infants now survive into adulthood [6]. As a result, clinicians will increasingly encounter adult patients who were born prematurely. Preterm birth should now be recognised as a chronic condition that predisposes to the development of diabetes across the life course. Physicians currently seldom seek birth histories from adult patients, and thus preterm birth may remain a ‘hidden’ risk factor. Medical records and history-taking in patients of all ages should routinely include birth history, including gestational age, birthweight and perinatal complications [4, 46–48]. Such information can help identify those born prematurely and facilitate anticipatory screening and early preventive actions, including patient counselling to promote lifestyle prevention of diabetes.

A key strength of the present study was the ability to examine gestational age at birth in relation to both type 1 and type 2 diabetes in a large national cohort with follow-up into adulthood, using birth, medical and pharmacy registry data that are highly complete. This study design minimises potential selection or ascertainment biases and enables more robust risk estimates based on a national population. The large sample size enabled well-powered assessment of narrowly defined gestational age groups and sex-specific differences. The results were controlled for other perinatal and maternal factors, as well as unmeasured familial factors using co-sibling analyses.

This study also had several limitations. First, laboratory data to verify diagnoses were unavailable. High positive predictive values have been reported for most chronic disorders in the Swedish registries, including diabetes (>99%) [23, 24]. However, to our knowledge, the validity of registry diagnoses for distinguishing type 1 and type 2 diabetes has not been evaluated. Our observed incidences were slightly lower than those previously reported based on serologic testing of autoantibodies and C-peptide to distinguish type 1 from type 2 diabetes in 1630 Swedish individuals of similar ages (e.g. type 1: 37.8 per 100,000 person-years at age 0–19 years vs 33.3 at age 0–18 years in the present study; type 2: 3.1 per 100,000 person-years at age 0–19 years vs 2.4 at age 0–18 years in the present study) [49]. It is possible that people born prematurely are more likely to be diagnosed with diabetes because of greater contact with the healthcare system (i.e. detection bias). However, this is most likely to affect relatively asymptomatic conditions early in life. Detection bias is less likely for type 1 diabetes because it is highly symptomatic, or in adulthood when most type 2 diabetes is diagnosed. Second, despite up to 43 years of follow-up, this was still a relatively young cohort. Additional follow-up will be needed to examine diabetes risks in older adulthood when such data become available in this or other large cohorts. Finally, this study was limited to Sweden and will need replication in other countries and diverse populations.

In summary, we found that preterm and early term birth were associated with increased risk of type 1 and type 2 diabetes from childhood into early to mid-adulthood in a large population-based cohort. Children and adults who were born prematurely may need early preventive evaluation and long-term follow-up for timely detection and treatment of diabetes.

## Electronic supplementary material

ESM Tables (PDF 206 kb)

## Data Availability

Owing to ethical concerns, supporting data cannot be made openly available. Further information about the data registries is available from the Swedish National Board of Health and Welfare: https://www.socialstyrelsen.se/en/statistics-and-data/registers/.

## Abbreviations

AGA: Appropriate for gestational age
LGA: Large for gestational age
SGA: Small for gestational age
