# Supplementary material for: Preterm birth and risk of type 1 and type 2 diabetes: a national cohort study
Source: Diabetologia. 2019 Dec 5;63(3):508–18. doi: 10.1007/s00125-019-05044-z (PMC6997251; doi:10.1007/s00125-019-05044-z)
Supplement: Supplementary file 1 — (PDF 206 kb) [file 125_2019_5044_MOESM1_ESM.pdf]

ELECTRONIC SUPPLEMENTARY MATERIAL

Contents

ESM Table 1. Unadjusted hazard ratios for type 1 and type 2 diabetes risk associated with gestational age at birth, Sweden, 1973-2015. Page 2

ESM Table 2. Interactions between gestational age at birth and sex in relation to type 1 diabetes risk. Page 3

ESM Table 3. Interactions between gestational age at birth and sex in relation to type 2 diabetes risk. Page 4

ESM Table 4. Co-sibling analyses for gestational age at birth in relation to type 1 and type 2 diabetes risk, Sweden, 1973-2015. Page 5

ESM Table 5. Associations between fetal growth and type 1 or type 2 diabetes risk, Sweden, 1973-2015. Page 6

ESM Table 6. Interactions between gestational age at birth and fetal growth in relation to type 1 diabetes risk. Page 7

ESM Table 7. Interactions between gestational age at birth and fetal growth in relation to type 2 diabetes risk. Page 8

ESM Table 1. Unadjusted hazard ratios for type 1 and type 2 diabetes risk associated with gestational age at birth, Sweden, 1973-2015.

	All			Females			Males		
	Cases	Rate[a]	HR (95% CI)[b]	Cases	Rate[a]	HR (95% CI)[b]	Cases	Rate[a]	HR (95% CI)[b]
**Attained ages <18 years**									
**Type 1 diabetes**									
Preterm (<37 wks)	1,177	40.59	1.26 (1.18, 1.33)	531	40.39	1.32 (1.20, 1.44)	646	40.75	1.20 (1.11, 1.30)
Extremely preterm (22-28 wks)	18	17.33	0.55 (0.34, 0.87)	7	14.16	0.47 (0.22, 0.98)	11	20.22	0.61 (0.34, 1.10)
Very preterm (29-33 wks)	172	31.52	0.98 (0.83, 1.14)	82	33.89	1.11 (0.89, 1.37)	90	29.64	0.88 (0.71, 1.08)
Late preterm (34-36 wks)	987	43.86	1.35 (1.27, 1.45)	442	43.20	1.41 (1.28, 1.55)	545	44.41	1.31 (1.20, 1.43)
Early term (37-38 wks)	4,060	38.31	1.19 (1.15, 1.23)	1,813	35.40	1.15 (1.10, 1.22)	2,247	41.04	1.21 (1.16, 1.27)
Full-term (39-41 wks)	13,610	32.36	Reference	6,360	30.72	Reference	7,250	33.95	Reference
Post-term (≥42 wks)	1,406	27.05	0.83 (0.79, 0.88)	617	25.68	0.83 (0.76, 0.90)	789	28.24	0.83 (0.77, 0.89)
Per additional week (trend)			0.95 (0.95, 0.96)			0.95 (0.94, 0.96)			0.95 (0.94, 0.96)
**Type 2 diabetes**									
Preterm (<37 wks)	89	3.07	1.43 (1.15, 1.78)	48	3.65	1.80 (1.33, 2.44)	41	2.59	1.15 (0.84, 1.58)
Extremely preterm (22-28 wks)	6	5.78	2.76 (1.23, 6.15)	4	8.09	4.07 (1.52, 10.88)	2	3.68	1.67 (0.42, 6.70)
Very preterm (29-33 wks)	25	4.58	2.14 (1.44, 3.19)	13	5.37	2.65 (1.53, 4.60)	12	3.95	1.76 (0.99, 3.13)
Late preterm (34-36 wks)	58	2.58	1.20 (0.92, 1.57)	31	3.03	1.49 (1.03, 3.15)	27	2.20	0.98 (0.66, 1.44)
Early term (37-38 wks)	328	3.10	1.45 (1.28, 1.65)	177	3.46	1.71 (1.43, 2.04)	151	2.76	1.23 (1.02, 1.48)
Full-term (39-41 wks)	901	2.14	Reference	421	2.03	Reference	480	2.25	Reference
Post-term (≥42 wks)	127	2.44	1.13 (0.94, 1.36)	46	1.91	0.93 (0.68, 1.26)	81	2.90	1.28 (1.01, 1.62)
Per additional week (trend)			0.93 (0.90, 0.95)			0.89 (0.86, 0.92)			0.97 (0.94, 1.01)
**Attained ages 18-43 years**									
**Type 1 diabetes**									
Preterm (<37 wks)	427	29.22	1.32 (1.20, 1.46)	168	25.77	1.41 (1.20, 1.65)	259	32.00	1.24 (1.09, 1.41)
Extremely preterm (22-28 wks)	19	49.66	2.22 (1.41, 3.48)	12	64.48	3.48 (1.97, 6.14)	7	35.62	1.36 (0.65, 2.87)
Very preterm (29-33 wks)	78	29.25	1.32 (1.06, 1.65)	28	23.89	1.30 (0.90, 1.89)	50	33.46	1.30 (0.98, 1.72)
Late preterm (34-36 wks)	330	28.54	1.29 (1.16, 1.45)	128	24.80	1.36 (1.14, 1.62)	202	31.55	1.23 (1.06, 1.41)
Early term (37-38 wks)	1,349	26.46	1.19 (1.12, 1.27)	511	21.39	1.16 (1.05, 1.28)	838	30.93	1.20 (1.11, 1.29)
Full-term (39-41 wks)	4,773	21.92	Reference	1,934	18.11	Reference	2,839	25.59	Reference
Post-term (≥42 wks)	710	21.93	1.02 (0.95, 1.11)	277	17.66	1.00 (0.88, 1.14)	433	25.96	1.03 (0.93, 1.14)
Per additional week (trend)			0.96 (0.95, 0.97)			0.95 (0.94, 0.97)			0.97 (0.95, 0.98)
**Type 2 diabetes**									
Preterm (<37 wks)	281	19.23	1.64 (1.45, 1.86)	142	21.78	1.96 (1.65, 2.33)	139	17.17	1.40 (1.17, 1.66)
Extremely preterm (22-28 wks)	11	28.75	2.73 (1.51, 4.93)	7	37.61	3.68 (1.75, 7.74)	4	20.36	1.87 (0.70, 5.00)
Very preterm (29-33 wks)	49	18.38	1.58 (1.19, 2.10)	30	25.60	2.33 (1.62, 3.34)	19	12.71	1.04 (0.66, 1.64)
Late preterm (34-36 wks)	221	19.11	1.62 (1.41, 1.86)	105	20.34	1.82 (1.49, 2.22)	116	18.12	1.47 (1.21, 1.77)
Early term (37-38 wks)	656	12.87	1.13 (1.04, 1.23)	308	12.89	1.19 (1.05, 1.35)	348	12.84	1.07 (0.95, 1.20)
Full-term (39-41 wks)	2,646	12.15	Reference	1,232	11.54	Reference	1,414	12.74	Reference
Post-term (≥42 wks)	497	15.35	1.13 (1.02, 1.24)	211	13.45	1.04 (0.90, 1.21)	286	17.14	1.20 (1.06, 1.36)
Per additional week (trend)			0.96 (0.94, 0.97)			0.93 (0.91, 0.95)			0.98 (0.96, 1.01)

[a]Incidence rate per 100,000 person-years
[b]Attained age was used as the Cox model time axis.

ESM Table 2. Interactions between gestational age at birth and sex in relation to type 1 diabetes risk at ages 0-43 years.

	Gestational age at birth						HRs (95% CI) for early term vs. full-term within sex strata	HRs (95% CI) for preterm vs. full-term within sex strata
	Full-term (39-41 wks)		Early term (37-38 wks)		Preterm (<37 wks)			
	Rate[a] (Cases)	HR (95% CI)[b]	Rate[a] (Cases)	HR (95% CI)[b]	Rate[a] (Cases)	HR (95% CI)[b]		
**Sex**								
Males	31.09 (10,089)	Reference	37.69 (3,085)	1.19 (1.14, 1.23); *P*<0.001	37.79 (905)	1.17 (1.09, 1.25); *P*<0.001	1.19 (1.14, 1.23); *P*<0.001	1.17 (1.09, 1.25); *P*<0.001
Females	26.43 (8,294)	0.85 (0.83, 0.87); *P*<0.001	30.94 (2,324)	0.96 (0.92, 1.01); *P*=0.09	35.54 (699)	1.09 (1.01, 1.18); *P*=0.03	1.13 (1.08, 1.18); *P*<0.001	1.28 (1.18, 1.38); *P*<0.001
HRs (95% CI) for females vs. males within gestational age strata		0.85 (0.83, 0.87); *P*<0.001		0.81 (0.77, 0.86); *P*<0.001		0.93 (0.84, 1.02); *P*=0.15		
Interaction on additive scale: RERI (95% CI)			-0.07 (-0.14, -0.01); *P*=0.02		0.07 (-0.04, 0.18); *P*=0.22			
Interaction on multiplicative scale: HR ratio (95% CI)			0.96 (0.90, 1.01); *P*=0.13		1.10 (0.98, 1.21); *P*=0.09			

[a]Incidence rate per 100,000 person-years

[b]Adjusted for child characteristics (birth year, birth order) and maternal characteristics (age, education, birth country or region, BMI, smoking, diabetes, preeclampsia, other hypertensive disorders during pregnancy)

HR = hazard ratio, RERI = relative excess risk due to interaction

ESM Table 3. Interactions between gestational age at birth and sex in relation to type 2 diabetes risk at ages 0-43 years.

	Gestational age at birth						HRs (95% CI) for early term vs. full-term within sex strata	HRs (95% CI) for preterm vs. full-term within sex strata
	Full-term (39-41 wks)		Early term (37-38 wks)		Preterm (<37 wks)			
	Rate[a] (Cases)	HR (95% CI)[b]	Rate[a] (Cases)	HR (95% CI)[b]	Rate[a] (Cases)	HR (95% CI)[b]		
**Sex**								
Males	5.84 (1,894)	Reference	6.10 (499)	1.05 (0.95, 1.16); *P*=0.33	7.52 (180)	1.19 (1.02, 1.39); *P*=0.02	1.05 (0.95, 1.16); *P*=0.33	1.19 (1.02, 1.39); *P*=0.02
Females	5.27 (1,653)	0.91 (0.85, 0.97); *P*=0.004	6.46 (485)	1.16 (1.05, 1.28); *P*=0.004	9.66 (190)	1.55 (1.33, 1.80); *P*<0.001	1.28 (1.15, 1.41); *P*<0.001	1.70 (1.45, 1.96); *P*<0.001
HRs (95% CI) for females vs. males within gestational age strata		0.91 (0.85, 0.97); *P*=0.004		1.10 (0.97, 1.24); *P*=0.14		1.30 (1.03, 1.56); *P*=0.03		
Interaction on additive scale: RERI (95% CI)			0.20 (0.05, 0.35); *P*=0.009		0.45 (0.16, 0.73); *P*=0.002			
Interaction on multiplicative scale: HR ratio (95% CI)			1.21 (1.04, 1.39); *P*=0.01		1.43 (1.12, 1.73); *P*=0.006			

[a]Incidence rate per 100,000 person-years
[b]Adjusted for child characteristics (birth year, birth order) and maternal characteristics (age, education, birth country or region, BMI, smoking, diabetes, preeclampsia, other hypertensive disorders during pregnancy)
HR = hazard ratio, RERI = relative excess risk due to interaction

ESM Table 4. Co-sibling analyses for gestational age at birth in relation to type 1 and type 2 diabetes risk, Sweden, 1973-2015.

Gestational age at birth	Type 1 diabetes		Type 2 diabetes	
	Cases	HR (95% CI)[a]	Cases	HR (95% CI)[a]
**Attained ages <18 years**				
Preterm (<37 wks)	214	1.16 (1.04, 1.30)	21	1.13 (0.75, 1.70)
Extremely preterm (22-28 wks)	3	0.68 (0.33, 1.38)	2	NE
Very preterm (29-33 wks)	38	0.90 (0.70, 1.15)	9	0.89 (0.40, 2.02)
Late preterm (34-36 wks)	173	1.24 (1.10, 1.39)	10	1.10 (0.70, 1.72)
Early term (37-38 wks)	577	1.09 (1.02, 1.16)	34	1.19 (0.96, 1.48)
Full-term (39-41 wks)	1,792	Reference	129	Reference
Post-term (≥42 wks)	212	0.96 (0.87, 1.05)	16	1.19 (0.86, 1.64)
Per additional week (trend)		0.97 (0.96, 0.99)		0.98 (0.93, 1.04)
**Attained ages 18-43 years**				
Preterm (<37 wks)	101	1.14 (0.92, 1.41)	93	1.55 (1.12, 2.13)
Extremely preterm (22-28 wks)	5	1.61 (0.59, 4.41)	7	2.66 (0.22, 31.75
Very preterm (29-33 wks)	22	1.26 (0.78, 2.06)	15	1.06 (0.55, 2.02)
Late preterm (34-36 wks)	74	1.10 (0.87, 1.38)	71	1.69 (1.19, 2.40)
Early term (37-38 wks)	271	1.05 (0.93, 1.17)	197	1.07 (0.89, 1.28)
Full-term (39-41 wks)	941	Reference	719	Reference
Post-term (≥42 wks)	150	1.09 (0.94, 1.26)	148	0.94 (0.75, 1.17)
Per additional week (trend)		0.99 (0.97, 1.02)		0.97 (0.93, 1.03)

[a]Adjusted for shared familial (genetic and/or environmental) factors in addition to specific child characteristics (birth year, sex, birth order) and maternal characteristics (age, education, birth country or region, BMI, smoking, diabetes, preeclampsia, other hypertensive disorders during pregnancy).

ESM Table 5. Associations between fetal growth and type 1 or type 2 diabetes risk, Sweden, 1973-2015.

	**Type 1 diabetes HR (95% CI)[a]**	**Type 2 diabetes HR (95% CI)[a]**
**Attained ages <18 years**		
SGA	0.88 (0.83, 0.93)	1.61 (1.38, 1.89)
AGA	Reference	Reference
LGA	1.09 (1.05, 1.14)	1.11 (0.94, 1.30)
**Attained ages 18-43 years**		
SGA	1.17 (1.09, 1.26)	1.79 (1.65, 1.93)
AGA	Reference	Reference
LGA	1.08 (1.00, 1.17)	0.95 (0.85, 1.07)

[a]Adjusted for child characteristics (birth year, sex, birth order) and maternal characteristics (age, education, birth country or region, BMI, smoking, diabetes, preeclampsia, other hypertensive disorders during pregnancy).
AGA = appropriate for gestational age, LGA = large for gestational age, SGA = small for gestational age

ESM Table 6. Interactions between gestational age at birth and fetal growth in relation to type 1 diabetes risk at ages 0-43 years.

	Gestational age at birth						HRs (95% CI) for early term vs. full-term within fetal growth strata	HRs (95% CI) for preterm vs. full-term within fetal growth strata
	Full-term (39-41 wks)		Early term (37-38 wks)		Preterm (<37 wks)			
	Rate[a] (Cases)	HR (95% CI)[b]	Rate[a] (Cases)	HR (95% CI)[b]	Rate[a] (Cases)	HR (95% CI)[b]		
**Fetal growth**								
AGA	28.55 (14,671)	Reference	33.45 (4,269)	1.15 (1.11, 1.19); *P*<0.001	35.78 (1,289)	1.20 (1.14, 1.27); *P*<0.001	1.15 (1.11, 1.19); *P*<0.001	1.20 (1.14, 1.27); *P*<0.001
SGA	26.70 (1,535)	0.99 (0.94, 1.05); *P*=0.77	30.93 (323)	1.11 (0.99, 1.24); *P*=0.06	30.52 (119)	1.04 (0.87, 1.24); *P*<0.69	1.12 (0.99, 1.25); *P*=0.08	1.05 (0.85, 1.24); *P*=0.65
HRs (95% CI) for SGA vs. AGA within gestational age strata		0.99 (0.94, 1.05); *P*=0.77		0.97 (0.86, 1.08); *P*=0.55		0.86 (0.70, 1.03); *P*=0.10		
Interaction on additive scale: RERI (95% CI)			-0.03 (-0.17, 0.11); *P*=0.66		-0.16 (-0.36, 0.05); *P*=0.13			
Interaction on multiplicative scale: HR ratio (95% CI)			0.97 (0.85, 1.10); *P*=0.68		0.87 (0.70, 1.04); *P*=0.13			

[a]Incidence rate per 100,000 person-years

[b]Adjusted for child characteristics (birth year, sex, birth order) and maternal characteristics (age, education, birth country or region, BMI, smoking, diabetes, preeclampsia, other hypertensive disorders during pregnancy)

AGA = appropriate for gestational age, HR = hazard ratio, RERI = relative excess risk due to interaction, SGA = small for gestational age

ESM Table 7. Interactions between gestational age at birth and fetal growth in relation to type 2 diabetes risk at ages 0-43 years.

	Gestational age at birth						HRs (95% CI) for early term vs. full-term within fetal growth strata	HRs (95% CI) for preterm vs. full-term within fetal growth strata
	Full-term (39-41 wks)		Early term (37-38 wks)		Preterm (<37 wks)			
	Rate[a] (Cases)	HR (95% CI)[b]	Rate[a] (Cases)	HR (95% CI)[b]	Rate[a] (Cases)	HR (95% CI)[b]		
**Fetal growth**								
AGA	5.12 (2,629)	Reference	5.66 (723)	1.14 (1.05, 1.23); *P*=0.002	7.52 (271)	1.41 (1.24, 1.59); *P*<0.001	1.14 (1.05, 1.23); *P*=0.002	1.41 (1.24, 1.59); *P*<0.001
SGA	10.58 (608)	1.76 (1.61, 1.92); *P*<0.001	12.45 (130)	2.17 (1.82, 2.59); *P*<0.001	13.33 (52)	2.24 (1.70, 2.96); *P*<0.001	1.24 (1.00, 1.47); *P*=0.05	1.28 (0.91, 1.64); *P*=0.14
HRs (95% CI) for SGA vs. AGA within gestational age strata		1.76 (1.61, 1.92); *P*<0.001		1.91 (1.55, 2.27); *P*<0.001		1.60 (1.12, 2.07); *P*=0.01		
Interaction on additive scale: RERI (95% CI)			0.28 (-0.13, 0.69); *P*=0.18		0.08 (-0.57, 0.73); *P*=0.81			
Interaction on multiplicative scale: HR ratio (95% CI)			1.09 (0.86, 1.31); *P*=0.44		0.91 (0.63, 1.19); *P*=0.52			

[a]Incidence rate per 100,000 person-years

[b]Adjusted for child characteristics (birth year, sex, birth order) and maternal characteristics (age, education, birth country or region, BMI, smoking, diabetes, preeclampsia, other hypertensive disorders during pregnancy)

AGA = appropriate for gestational age, HR = hazard ratio, RERI = relative excess risk due to interaction, SGA = small for gestational age
